# A Human-Algorithm Integration System for Hip Fracture Detection on Plain Radiography: System Development and Validation Study

**DOI:** 10.2196/19416

**Published:** 2020-11-27

**Authors:** Chi-Tung Cheng, Chih-Chi Chen, Fu-Jen Cheng, Huan-Wu Chen, Yi-Siang Su, Chun-Nan Yeh, I-Fang Chung, Chien-Hung Liao

**Affiliations:** 1 Department of Trauma and Emergency Surgery Linkou Chang Gung Memorial Hospital Chang Gung University Taoyuan Taiwan; 2 Department of Physical Medicine and Rehabilitation Linkou Chang Gung Memorial Hospital Chang Gung University Taoyuan Taiwan; 3 Department of Emergency Medicine Kaohsiung Chang Gung Memorial Hospital Chang Gung University Taoyuan Taiwan; 4 Department of Medical Imaging & Intervention Linkou Chang Gung Memorial Hospital Chang Gung University Taoyuan Taiwan; 5 Department of General Surgery Linkou Chang Gung Memorial Hospital Chang Gung University Taoyuan Taiwan; 6 Institute of Biomedical Informatics National Yang-Ming University Taipei Taiwan; 7 Center for Systems and Synthetic Biology National Yang-Ming University Taipei Taiwan; 8 Preventive Medicine Research Center Taipei Taiwan

**Keywords:** hip fracture, neural network, computer, artificial intelligence, algorithms, human augmentation, deep learning, diagnosis

## Abstract

**Background:**

Hip fracture is the most common type of fracture in elderly individuals. Numerous deep learning (DL) algorithms for plain pelvic radiographs (PXRs) have been applied to improve the accuracy of hip fracture diagnosis. However, their efficacy is still undetermined.

**Objective:**

The objective of this study is to develop and validate a human-algorithm integration (HAI) system to improve the accuracy of hip fracture diagnosis in a real clinical environment.

**Methods:**

The HAI system with hip fracture detection ability was developed using a deep learning algorithm trained on trauma registry data and 3605 PXRs from August 2008 to December 2016. To compare their diagnostic performance before and after HAI system assistance using an independent testing dataset, 34 physicians were recruited. We analyzed the physicians’ accuracy, sensitivity, specificity, and agreement with the algorithm; we also performed subgroup analyses according to physician specialty and experience. Furthermore, we applied the HAI system in the emergency departments of different hospitals to validate its value in the real world.

**Results:**

With the support of the algorithm, which achieved 91% accuracy, the diagnostic performance of physicians was significantly improved in the independent testing dataset, as was revealed by the sensitivity (physician alone, median 95%; HAI, median 99%; *P*<.001), specificity (physician alone, median 90%; HAI, median 95%; *P*<.001), accuracy (physician alone, median 90%; HAI, median 96%; *P*<.001), and human-algorithm agreement [physician alone κ, median 0.69 (IQR 0.63-0.74); HAI κ, median 0.80 (IQR 0.76-0.82); *P*<.001. With the help of the HAI system, the primary physicians showed significant improvement in their diagnostic performance to levels comparable to those of consulting physicians, and both the experienced and less-experienced physicians benefited from the HAI system. After the HAI system had been applied in 3 departments for 5 months, 587 images were examined. The sensitivity, specificity, and accuracy of the HAI system for detecting hip fractures were 97%, 95.7%, and 96.08%, respectively.

**Conclusions:**

HAI currently impacts health care, and integrating this technology into emergency departments is feasible. The developed HAI system can enhance physicians’ hip fracture diagnostic performance.

## Introduction

Deep learning (DL) is a subset of machine learning that uses an advanced form of artificial neural networks; the use of DL has impacted health care [1,2]. Numerous applications of DL in medicine, such as computer-aided diagnosis, have been studied [3-9].

Several studies have shown the possibility of using algorithms trained with a large amount of data to aid in appropriate triage, accurately predicting outcomes, improving diagnoses and referrals in clinical situations, and even shortening the waiting time for reports [10-15]. An increasing amount of supporting evidence shows that the use of computer vision with deep neural networks—a rapidly advancing technology ideally suited to solving image-based problems—achieves excellent performance, comparable to that of experts [12-18].

An increasing number of studies have reported the influence of DL in health care, from its use in pathological evaluation to radiographic image assessment [8,9,17,18]. These reports help us understand DL algorithm behavior and how to apply algorithms to reduce medical costs, facilitate further preventive practices, and increase the quality of health care [3].

Hip fractures are among the leading fracture types in elderly individuals worldwide and are the cause of yearly increases in medical costs [19-22]. At the first hospital evaluation, 4-14% of patients’ diagnoses are missed [23-25]. Pelvic radiographs (PXRs) are the first-line imaging modality; however, there is a risk of low sensitivity and missed diagnoses when only the image is read. The efficacy and efficiency of several algorithms for skeletal radiology, including hip fracture recognition, have been proven [18,26-28]. However, current state-of-the-art applications of DL to plain film reading by first-line physicians have not been integrated into practice.

Most medical image studies have compared a trained DL algorithm with the performance of human specialists with the goal of developing an algorithm that can outperform specialists [14,26,27,29]. However, there is a lack of studies assessing the combined performance of physician judgment and algorithm prediction, which is a possible situation in clinical scenarios. In this study, we developed a human-algorithm integration (HAI) system to detect hip fractures. Furthermore, we incorporated the HAI system into clinical workflows to improve diagnostic efficiency and accuracy.

## Methods

### Materials

We utilized data from the Chang Gung Trauma Registry Programme (CGTRP) from Chang Gung Memorial Hospital (CGMH), Linkou, Taiwan. Demographic data, medical records, medical imaging, and associated medical information were recorded prospectively in a computerized database. We extracted the data and images of all trauma patients treated between August 2008 and December 2017 at CGMH, which is a level 1 trauma center. The Internal Review Board of CGMH approved this study. Details of the dataset and image collection process are described in Multimedia Appendix 1.

### Development of the Hip Fracture Detection Algorithm and Algorithm Validation

**Figure 1:**
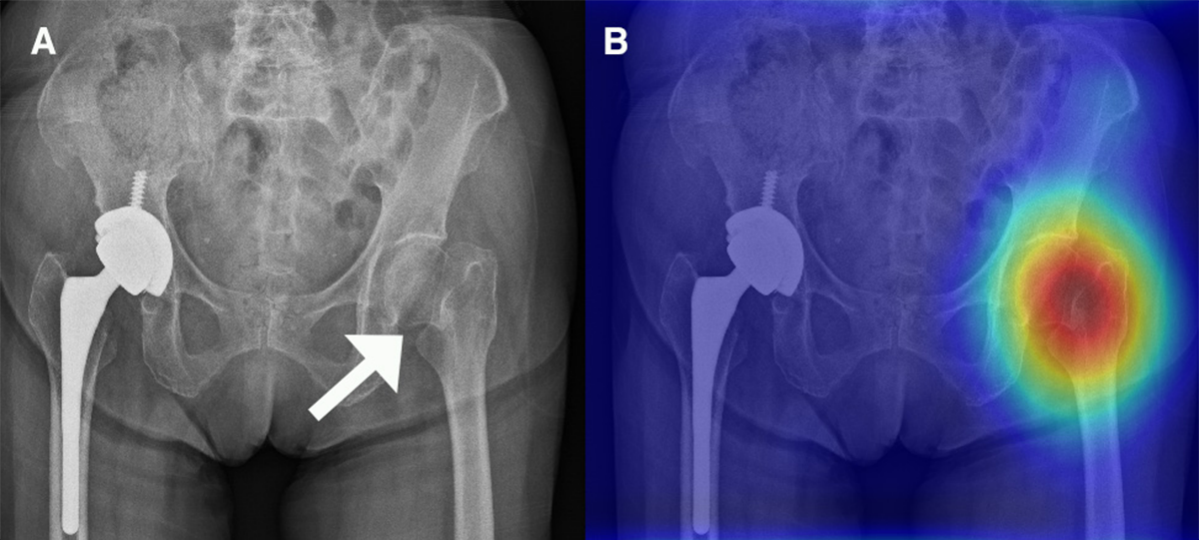
(A) Pelvic x-ray (PXR) with hip fracture. (B) PXR with left hip fracture with a human-algorithm integration–enhanced reference image.

The development of the hip fracture detection algorithm is based on a previous work [18] and described in detail in Multimedia Appendix 1. In summary, we obtained 3605 PXRs, which included 1975 films with hip fractures and 1630 films without hip fractures, from CGMH in Linkou, Taiwan, between August 2008 and December 2016. The diagnostic standard was based on all the available clinical information, including clinical diagnosis, imaging reports, advanced imaging reports, and operative findings. We randomly separated the development dataset into training (2163/3605, 60%), validation (721/ 3605, 20%), and testing (721/3605, 20%) sets for the initial evaluation of the performance of each neural network in hip fracture classification. We assessed VGG16, ResNet-152, Inception-v3, Inception-ResNet-v2, and DenseNet-121 with binary classification with randomly initialized weights. The DenseNet-121 [30] model showed balanced performance regarding the training, validation, and testing sets. Therefore, DenseNet-121 was selected for the classification structure of the deep convolutional neural network (DCNN). We also created heatmaps with gradient-weighted class activation mapping (Grad-CAM) [31] for fracture site detection. We applied the Adam optimizer with an initial learning rate of 10^-3^. The batch size was 8, and the DCNN was trained for 60 epochs without early stopping. This algorithm was able to evaluate the PXRs and generate a probability of hip fracture and a heatmap overlay for the original image to highlight the possible fracture area, generating an algorithm-assisted reference image for clinician use (Figure 1).

An independent dataset of 100 PXRs (50 hip fracture films and 50 films without hip fractures) from 2017 was collected to evaluate the performance of the algorithm. We set the probability threshold to 0.5, and a cut-off value was also applied in this study. This dataset was used to test the performance of the HAI system.

### Study Population, Physician, and the HAI System Performance Test

We enrolled certified medical doctors with different subspecialties and levels of experience and then assessed their performance in an image reading task to validate the HAI system’s performance. Subgroup analyses were also performed according to the physicians’ experience levels and specialties. Physicians who care for patients in the trauma bay were considered primary physicians, and those who treat patients after consultation (orthopedic surgeons and radiologists) were considered consulting physicians. Based on experience, physicians who had practiced for more than 3 years composed the experienced group; the other physicians composed the novice group.

**Figure 2:**
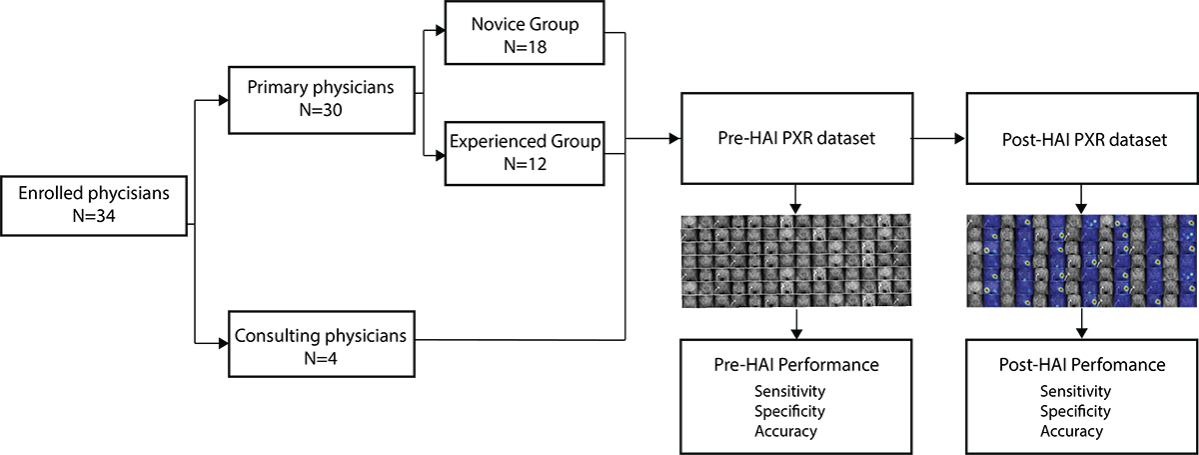
The validation flow of the human-algorithm integration (HAI) system performance test. PXR: plain pelvic radiograph.

Before the examination, we introduced the physicians to the study design and provided the image collection details. Figure 2 shows the validation flow of the HAI performance test.

The physicians examined the dataset of 100 PXRs at their original resolution (50 images with fractures and 50 images without fractures) from the validation set from 2017. The physicians were able to zoom in on the images. Upon reviewing the dataset, the physicians were asked to diagnose the presence of a hip fracture. The sensitivity, specificity, and accuracy values obtained composed the physician-alone performance values. Then, the physicians assessed another randomly ordered set of 100 PXRs from the same dataset but with the algorithm-produced reference images, and they were asked to diagnose the presence of a hip fracture. We examined the sensitivity, specificity, and accuracy values of the physicians’ HAI performance. We compared the differences in the sensitivity, specificity, and accuracy values based on the algorithm only, the physician’s readings only, and the HAI combination. The agreement between the physicians and the algorithm was also calculated on the physician alone and the HAI data.

### Real-World Data Study

**Figure 3:**
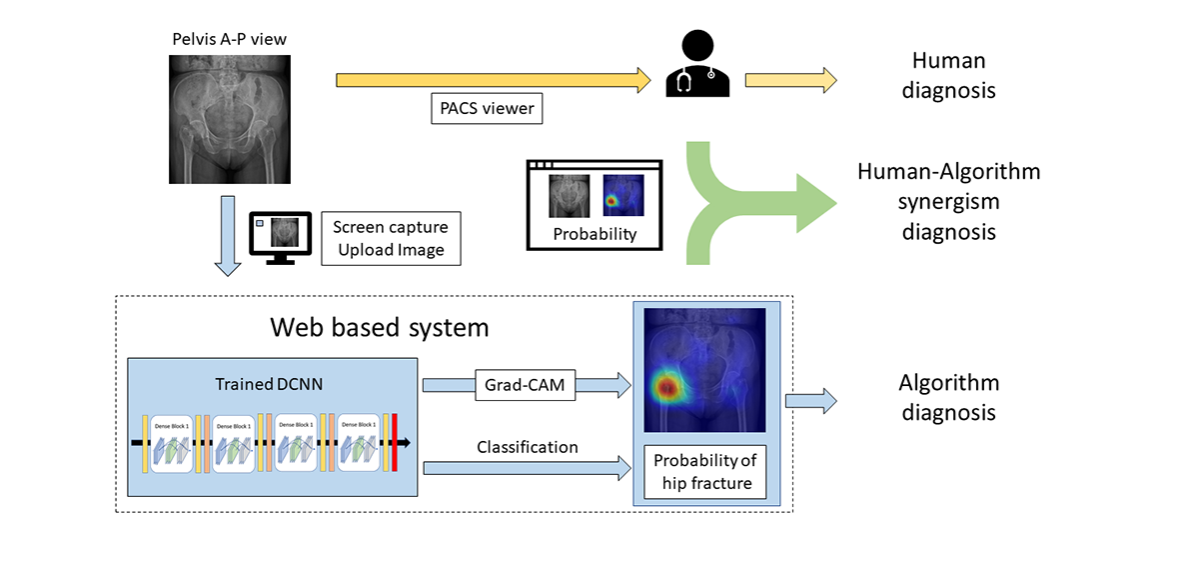
The flow of clinical integration of the human-algorithm integration (HAI) system into the emergency department and real-world data validation. DCNN: deep convolutional neural network; Grad-CAM: gradient-weighted class activation mapping; PACS: picture archiving and communication system.

After validating the feasibility and efficacy of the HAI system, we incorporated the HAI system for physician use in trauma bays and emergency departments at 3 trauma centers in Taiwan: Taipei CGMH, Linkou CGMH, and Kaohsiung CGMH. The physicians could initiate the inference platform of the HAI system while reviewing an image in the picture archiving and communication system (PACS) viewer. The HAI system captured and cropped the PXR from the PACS viewer and transferred it to the central server; the probability of hip fracture was calculated and presented to the clinical physicians along with the original PXR and the reference image (Figure 3).

All of the images received feedback from the clinical physician to validate whether the diagnosis from the HAI system was correct, and the data were recorded on the server. From the physician's feedback and the clinical diagnosis, we obtained the final report of the accuracy, sensitivity, and specificity of the HAI system. The gold-standard hip fracture diagnosis was the final diagnosis based on all the available clinical information.

### Statistical Analysis and Software

The DCNN was built and applied on a machine equipped with the Ubuntu 14.04 operation system (Canonical) with TensorFlow 1.5.1 (Google Brain), Keras 2.1.4, and Keras-vis 0.4.1. Statistical analysis and plots were performed in R 3.4.4 (Microsoft) with the ggplot2 (version 2.0.0; Hadley Wickham) and irr (version 0.84.1; Matthias Gamer et al) packages. Continuous variables were compared using Mann-Whitney U tests and Kruskal-Wallis tests, and categorical variables were evaluated with chi-squared tests. We evaluated the physician-alone and the HAI performance using the sensitivity, specificity, false-negative rate, false-positive rate, and F1 scores; 95% confidence intervals (CIs) were also calculated. Nonnormally distributed data are expressed as medians and interquartile ranges (IQRs). Agreement between the physician and the algorithm was calculated with Cohen kappa. The physician-alone performance and the HAI performance were compared using Wilcoxon signed-rank tests. Receiver operating characteristic (ROC) curves and the areas under the ROC curves (AUCs) were used to evaluate the performance of the model.

## Results

### DL Algorithm Performance

After applying the hip model to the testing dataset (n=100, normal=50, fractures=50), the sensitivity, specificity, accuracy, and false-negative rate of the model were 98% (95% CI 89%-100%), 84% (95% CI 71%-93%), 91% (n=100; 95% CI 84%-96%), and 2% (95% CI 0.3%-17%), respectively.

### Physician and HAI Performance

In total, 34 physicians with a median practice time of 4 (IQR 3.0-5.0) years, including 4 consulting physicians (2 radiologists and 2 orthopedic surgeons) and 30 primary physicians [21 surgeons, 6 emergency physicians, and 3 postgraduate-year (PGY) doctors], completed the examination, as shown in Table 1.

**Table 1:** Demographic data of the physician participants (n=34).

Physician characteristics		Values
Age in years, median (IQR)		29.00 (27.00-32.00)
Years of practice, median (IQR)		3.00 (2.00-5.75)
**Gender, n (%)**		
	Male	27 (79)
	Female	7 (21)
**Physician subspecialties, n (%)**		
	General surgeon	21 (62)
	Emergency physician	6 (18)
	Postgraduate-year doctor	3 (9)
	Radiologist	2 (6)
	Orthopedic surgeon	2 (6)

Table 2 shows that the median sensitivity of the primary physicians in the physician-alone testing was 95% (IQR 90%-100%), the median specificity was 90% (IQR 82%-94%), and the median accuracy was 90% (IQR 88%-94%). The median kappa between the physicians and the algorithm was 0.69 (95% CI 0.63-0.74).

**Table 2:** The physician-alone performance and human-algorithm integration (HIA) performance of the physician participants (n=34); the Wilcoxon signed-rank test was used to compare the physician-alone and the HAI performance.

Measures	Physician-alone performance	HAI performance	*P* value^a^
Sensitivity, median (IQR)	0.95 (0.90-1.00)	0.99 (0.96-1.00)	<.001
Specificity, median (IQR)	0.90 (0.82-0.94)	0.95 (0.90-0.98)	<.001
Accuracy, median (IQR)	0.90 (0.88-0.94)	0.96 (0.93-0.98)	<.001
Human-algorithm agreement, κ, median (IQR)	0.69 (0.63-0.74)	0.80 (0.76-0.82)	<.001

^a^All *P* values are statistically significant.

**Figure 4:**
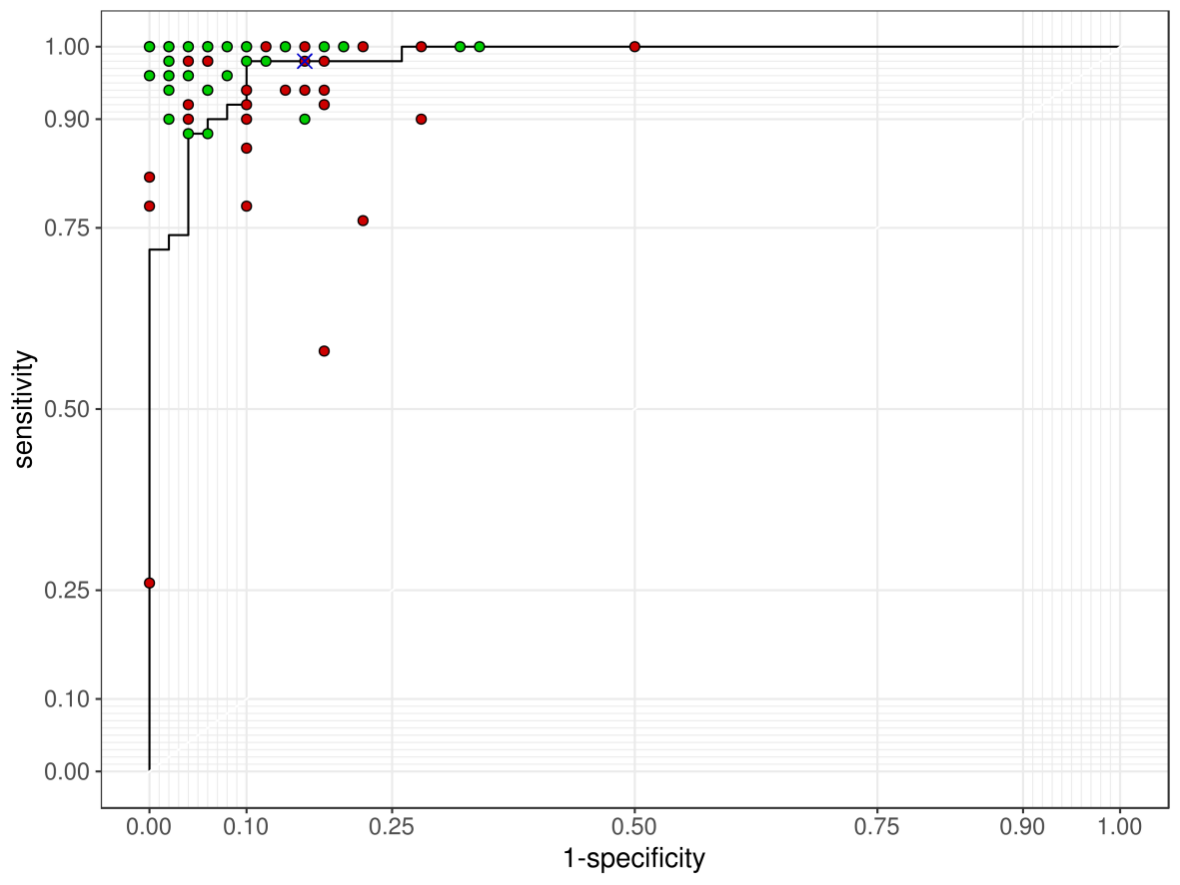
The receiver operating characteristic curve of the algorithm performance on test images. Green spots: participants’ performance; red spots: participants’ performance with human-algorithm integration (HAI) assistance; cross mark: the cut-off performance of the algorithm presented to the physician.

After the HAI system was applied, the median sensitivity of the HAI system was 99% (IQR 96%-100%), the median specificity was 95% (IQR 90%-98%), and the median accuracy was 96% (IQR 93%-98%). The median kappa between the physicians and the algorithm was 0.80 (IQR 0.76-0.82). All of the above factors were significantly improved after HAI system implementation (Table 2). Most of the physicians’ performances improved after the algorithm-assisted test, as shown in Figure 4.

**Figure 5:**
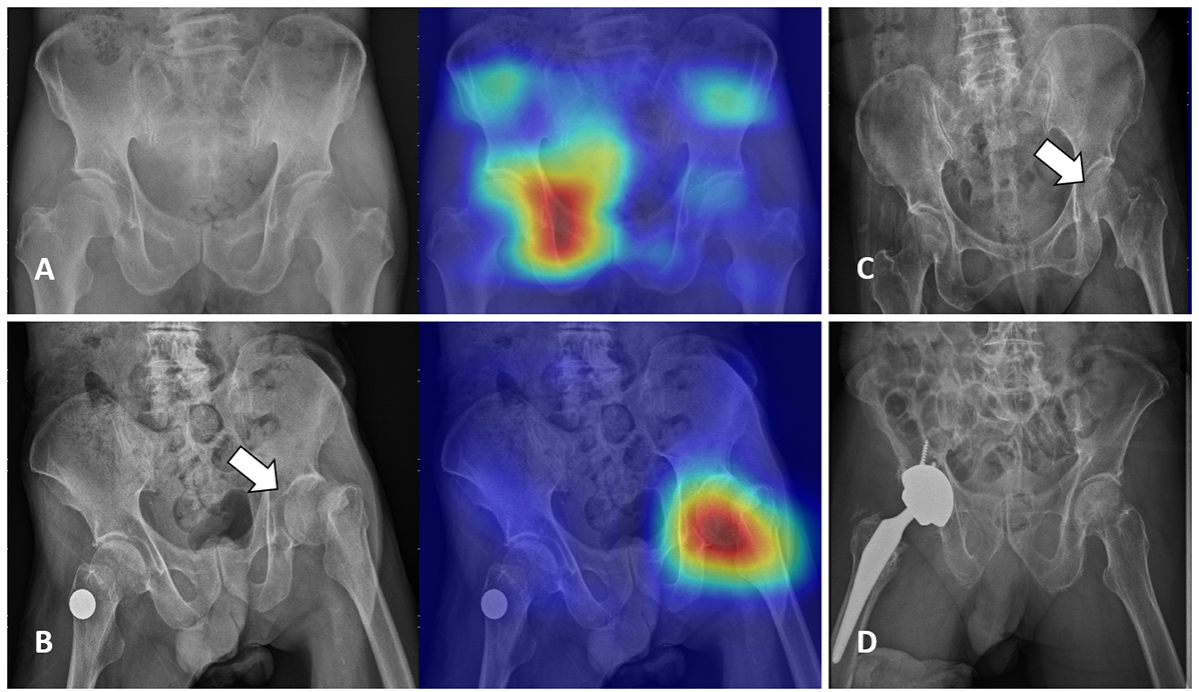
Examples of inconsistencies between the participants and the algorithm. (A) A pelvic x-ray (PXR) without hip fracture that was overdiagnosed by the algorithm. No participant overdiagnosed in the physician-alone test, and only 1 (2.9%) participant overdiagnosed in the human-algorithm integration (HAI) test. (B) A PXR with left hip fracture. In the physician-alone test, 12 (35.3%) participants missed this fracture. In the HAI test, only 1 (2.9%) participant missed this fracture. (C) A PXR with left hip fracture that was missed by the algorithm. In the physician-alone test, 4 (11.8%) participants missed this fracture. In the HAI test, 3 (8.8%) participants missed this fracture. (D) A PXR without hip fracture. In the physician-alone test, 18 (52.9%) participants overdiagnosed this image. In the HAI test, only 5 (14.7%) participants overdiagnosed this image.

The agreement between the physicians and the algorithm also increased but was still not entirely consistent. Among all the HAI system results, the algorithm had a false-positive rate of 8% per questionnaire, compared with a false-positive rate of only 0.91% per questionnaire for the physicians plus the algorithm. On the other hand, 3.76% of the fractures per questionnaire that were not identified on the physician-alone test were correctly identified after the algorithm information was provided, as shown in Figure 5.

Furthermore, the results were divided according to the physicians’ specialties, as shown in Table 3. The consulting physicians achieved a better performance than the primary physicians. Regarding the HAI performance, although there were still significant differences in the overall accuracy between specialties, there were no significant differences in the sensitivity and specificity.

**Table 3:** The physician-alone performance and human-algorithm integration (HAI) performance of the physicians by specialty (n=34).

Physician characteristics and performance		Primary physicians			Consulting physicians		*P* value
		General surgeons (n=21)	Emergency-department physicians (n=6)	Postgraduate-year physicians (n=3)	Radiologists (n=2)	Orthopedic surgeons (n=2)	
Age in years, median (IQR)		28.00 (27.00-30.00)	33.50 (29.75-37.25)	26.00 (25.50- 26.50)	39.00 (36.00-42.00)	33.50 (32.75-34.25)	.006^a^
Years of experience, median (IQR)		3.00 (2.00- 4.00)	5.50 (2.75-9.00)	1.00 (1.00- 1.00)	6.50 (6.25- 6.75)	12.50 (10.75- 14.25)	.003^a^
**Physician-alone performance**							
	Human-algorithm agreement, κ, median (IQR)	0.69 (0.63-0.72)	0.75 (0.67- 0.80)	0.44 (0.32- 0.53)	0.79 (0.78- 0.79)	0.72 (0.71- 0.74)	.027^a^
	Accuracy, median (IQR)	0.90 (0.88- 0.92)	0.95 (0.91- 0.97)	0.70 (0.66- 0.76)	0.96 (0.96- 0.97)	0.94 (0.93- 0.94)	.013^a^
	Sensitivity, median (IQR)	0.94 (0.90-0.98)	1.00 (0.98- 1.00)	0.58 (0.42- 0.74)	0.99 (0.98- 0.99)	1.00 (1.00- 1.00)	.003^a^
	Specificity, median (IQR)	0.90 (0.82- 0.96)	0.91 (0.83- 0.94)	0.82 (0.77- 0.91)	0.94 (0.94- 0.94)	0.87 (0.85- 0.88)	.855
**HAI performance**							
	Human-algorithm agreement, κ, median (IQR)	0.80 (0.76- 0.82)	0.81 (0.78- 0.83)	0.76 (0.74- 0.78)	0.78 (0.76- 0.80)	0.82 (0.82- 0.82)	.496
	Accuracy, median (IQR)	0.95 (0.94- 0.97)	0.98 (0.97- 0.99)	0.91 (0.89- 0.91)	0.97 (0.96- 0.97)	1.00 (1.00- 1.00)	.011^a^
	Sensitivity, median (IQR)	0.98 [0.96, 1.00]	1.00 (1.00- 1.00)	0.90 (0.89- 0.95)	0.97 (0.95- 0.98)	1.00 (1.00- 1.00)	.121
	Specificity, median (IQR)	0.94 (0.90-0.98)	0.97 (0.94- 0.98)	0.84 (0.83- 0.89)	0.97 (0.96- 0.97)	1.00 (1.00- 1.00)	.071

^a^*P* value is statistically significant.

To evaluate the influence of the physicians’ clinical experience on the use of the HAI system, we divided the primary physicians into novice and experienced groups, and the results are shown in Table 4. The experienced physicians showed a significantly higher sensitivity and slightly lower specificity than the novice physicians. After the integration of the algorithm information, the overall performance increased regardless of clinical experience.

**Table 4:** A comparison of the primary physician performance with the human-algorithm integration (HAI) performance, divided by physician experience (n=30); the Wilcoxon signed-rank test was used to compare the physician-alone performance and the HAI performance.

Primary physician characteristics and performance			Novice group (n=18)		Experienced group (n=12)		*P* value	
Age in years, median (IQR)			27.00 (27.00-28.00)		32.00 (30.75-34.25)		<.001^a^	
Years of experience, median (IQR)			2.00 (2.00-3.00)		5.00 (4.00-6.25)		< .001^a^	
**Performance evaluation**								
	**Human-algorithm agreement, κ, median (IQR)**							
		Physician alone		0.66 (0.62-0.72)		0.69 (0.64-0.77)		.330
		HAI		0.77 (0.71-0.80)		0.82 (0.79-0.82)		.008^a^
		Paired test, *P* value		.0001^a^		.001^a^		
	**Accuracy, median (IQR)**							
		Physician alone		0.90 (0.82-0.92)		0.90 (0.89-0.96)		.279
		HAI		0.94 (0.91-0.97)		0.97 (0.95-0.98)		.020 ^a^
		Paired test, *P* value		.0023^a^		.0032^a^		
	**Sensitivity, median (IQR)**							
		Physician alone		0.91 (0.83-0.95)		0.98 (0.94-1.00)		.017^a^
		HAI		0.97 (0.94-1.00)		1.00 (0.97-1.00)		.043^a^
		Paired test *P* value		.0028^a^		.0313^a^		
	**Specificity, median (IQR)**							
		Physician alone		0.89 (0.84-0.94)		0.86 (0.81-0.94)		.733
		HAI		0.94 (0.88-0.96)		0.96 (0.92-0.98)		.215
		Paired test, *P* value		.1067		.0049^a^		

^a^*P* value is statistically significant.

### Real-World Validation of the HAI System

In total, 632 tests were completed between March 24, 2019, and August 3, 2019. Images were excluded for the following reasons: (1) poor quality, (2) incorrect image input, such as chest plain film or computed tomography (CT), and (3) PXRs of pediatric patients. After excluding images for the above reasons, 587 PXRs qualified for inclusion. Among the 587 PXRs, there were 320 normal PXRs and 267 PXRs that showed hip fractures. The algorithm’s diagnostic accuracy was 92.67% (95% CI 90.26%-94.65%), the sensitivity was 91.01% (95% CI 86.92%-94.16%), the specificity was 94.06% (95% CI 90.88%-96.39%), and the false-negative rate was 7.33%. Of the 587 PXRs, the physicians’ diagnoses were consistent with the algorithm for 561 images (95.57%) and were inconsistent for 26 images (4.43%). After reference image assistance, the diagnostic accuracy of the HAI system was 97.10% (95% CI 95.40%-98.30%), the sensitivity was 99.25% (95% CI 97.32%-99.91%), and the specificity was 95.31% (95% CI 92.39%-97.35%). Of the 587 images, 2 images could not be diagnosed by the HAI system; these 2 patients required a CT for hip fracture diagnosis. The false-negative rate of the HAI system was 0.65%, as presented in Table 5.

**Table 5:** Clinical validation of the human-algorithm integration (HAI) system in emergency departments.

Algorithm-only vs. HIA diagnosis		Fracture		Sensitivity, % (95% CI)		Specificity, % (95% CI)		Accuracy, % (95% CI)	
		(+)	(–)						
**Algorithm-only diagnosis**				91.01 (86.92%-94.16%)		94.06 (90.88%-96.39%)		92.67 (90.26%-94.65%)	
	(+)	243	19						
	(–)	24	301						
**HAI diagnosis**				99.25 (97.32%-99.91%)		95.31 (92.39%-97.35%)		97.10 (95.40%-98.30%)	
	(+)	265	15						
	(–)	2	305						
						

## Discussion

In this study, we demonstrated 2 findings. First, the HAI system, which integrates an algorithm and human intelligence, performed better than the physicians alone and the algorithm alone. Second, we integrated the HAI system into the clinical flow and verified its use in real-world trauma bays. For orthopedic radiology, fracture detection with computed-aid diagnosis is one of the first applications of AI in radiologic imaging [32,33]. In this study, with the assistance of the HAI system, the physicians detected hip fractures with an increased diagnostic accuracy ranging from 2% to 22%. Several studies demonstrate the strong performance of DL algorithms for fracture detection from different anatomic sites, such as the wrist, humeral, foot, and femur. In this study, our algorithm performance was not inferior to these previous results [26-28]. Furthermore, previous studies usually compared the results of an algorithm with those of professional personnel or other algorithms [17,26,28,34,35].

In the current environment, the algorithm does not replace human intelligence, especially in health care; however, a DL algorithm can complement and augment the ability and knowledge of physicians [1,36,37]. Until now, no real-world data from clinical studies have shown that the integration of AI into the clinical environment can aid physicians. Our study provides the first evidence that HAI can assist patients and doctors in the trauma bay, and we have demonstrated the feasibility of an HAI system to increase diagnostic accuracy.

Some issues occur with the use of computer-assisted diagnostic tools [38,39]. First, the algorithm makes decisions based on features that need to be explored, and there are inevitable caveats, even though the predictions may be correct. Another issue is that physicians may overly rely on the algorithm and disregard their own judgment. To resolve these issues, the HAI system offers physicians a heatmap that highlights the probable location of the fracture on the reference PXR, thus helping physicians understand how the algorithm works. The physician needs to review the image and make the final diagnosis, which prevents him or her from over-relying on the HAI system. We designed a method integrating human expertise and computers that fits the clinical context [38-40], and the HAI system can increase the diagnostic accuracy and specificity. After implementing the validation test performed by 34 physicians, we found that the HAI system performed better than the physicians alone and the algorithm alone (accuracy: 90% vs. 86% vs. 90%; false-negative rate: 6% vs. 12% vs. 9%, respectively). Moreover, we found that with the HAI system, novice physicians can increase their diagnostic accuracy to more closely approach that of experienced physicians, and even consulting physicians.

Machine learning methods have a tendency to “overfit” to idiosyncrasies in the training sample, which may yield overly optimistic performance estimates [36,37]. When addressing the challenges of clinical usage, another question arises: Can the DL algorithm handle real-world data in addition to edited information [1]? Limited studies have proven that algorithms can be applied to real-world data [14], but the clinical effects are still being evaluated. In this study, we have proved that HAI helps physicians detect hip fractures. We operated the HAI system in the trauma bays of 3 trauma centers and obtained adequate hip fracture recognition results. In a real-world validation study, the HAI system improved the accuracy of hip fracture diagnosis to 97%, with a false-negative rate of 0.65%. Several reports have shown that the algorithm might help physicians in acute care and could save lives [10-15].

We did not develop an excellent complete AI solution that can address all situations in health care. However, with the support of HAI, we can reduce some preventable costs and functional losses in fragile fracture cases, improve the allocation of resources, reduce the need for unnecessary consultations, and facilitate faster patient disposition [1,3]. HAI has the potential to improve the delivery of efficient and high-quality care in high-volume clinical practice while allowing physicians to focus on more conceptually demanding tasks by offloading their more mundane duties [3,41].

The clinical usage of the proposed HAI system can improve diagnostic accuracy and reduce the unnecessary use of CT. However, there are still some limitations. First, because we defined our system as an HAI system, selection bias might exist, and clinical physicians could always use this tool when they were unsure about the presence of a fracture. Therefore, some of the images may have been excluded. Second, PXRs provide information on not only skeletal fractures but also soft tissue changes. Although our HAI system can detect fracture sites on PXRs, it still lacks information needed to detect other lesions and cannot replace the expertise of radiologists and clinical physicians. Third, the HAI system does not currently integrate clinical information, which differs from the considerations of clinical practice. The integration of clinical data into the HAI system is another challenge [42]. Fourth, the limited number of physicians participating in this evaluation might have resulted in an underpowered study. Fifth, the images of the validation dataset are only from one institute, which might induce selection bias as well. Finally, a preliminary study of 600 testing images was performed. However, this study tested a limited number of cases at 3 different trauma centers, which are further limitations. The future development of a prospective multicenter study should be used to investigate the system’s function in the real world.

In conclusion, the HAI system improves diagnostic accuracy, and the integration of this technology into the clinical flow is feasible. The HAI system can enhance the performance of physicians, especially novice clinicians.

## Appendix

Multimedia Appendix 1 The dataset and algorithm development of the Human-Algorithm Integration (HAI) system for hip fracture.

## Abbreviations

AUC: area under the curve
CT: computed tomography
DCNN: deep convolutional neural network
DL: deep learning
Grad-CAM: gradient-weighted class activation mapping
HAI: human-algorithm integration
PXR: pelvic radiograph
ROC: receiver operating characteristic
