# Supplementary material for: A Human-Algorithm Integration System for Hip Fracture Detection on Plain Radiography: System Development and Validation Study
Source: JMIR Med Inform. 2020 Nov 27;8(11):e19416. doi: 10.2196/19416 (PMC7732715; doi:10.2196/19416)
Supplement: Multimedia Appendix 1 [file medinform_v8i11e19416_app1.docx]

**Supplementary Materials**

**Supplementary method**

1. **Dataset**
2. **Image collection process and deidentification**
3. **Pre-training dataset**
4. **Development and testing dataset**
5. **Annotation of the development and testing dataset**
6. **Algorithm development**
7. **Platform and hardware**
8. **Image preprocessing and augmentation**
9. **Neural network architecture selection**
10. **Training process of the algorithm**

**Supplementary references**

1. **Dataset**
   1. **Image collection process and deidentification**The X-ray images used in this study are collected from the PACS system of the Chang Gung Memorial Hospital, Linkou. All the trauma patient admitted to the hospital is recorded in the trauma registry with basic demographic information, diagnosis description, diagnosis ICD-9 code, injury severity score, treatment course, and outcome. We extracted the patient list and performed a radiography search in the PACS repository. The image on the date of injury was collected and converted into PNG format with 8-bit grayscale color. The type of image including ankle, foot, knee, wrist, elbow, and pelvis X-ray. The chart number and any tags revealing the patient identity on the images were removed by cropping. The collected images size ranges from 2128 × 2248 pixels to 2688 × 2688 pixels.
   2. **Pre-training dataset**

The limb dataset includes 6019 ankle, 4134 foot, 8142 knee, 3378 wrist, and 3832 elbow radiographs. The limb dataset was used for pre-training with only body part label corresponding to each image.

- 1. **Development and testing dataset**

The development dataset includes 3605 pelvic radiographs (PXR) obtained from Aug 2008 to Dec 2016. The dataset contains 1975 hip fracture images with 931(47.1%) femoral neck fractures and 1044(52.9%) trochanteric fractures. The mean age of hip fracture patients is 72.34±16.73, with 42% male. We also collected 1630 PXRs without a hip fracture from the trauma registry. The mean age of patients without hip fracture is 44.88±20.46, with 68.2% male.

The independent testing set was randomly selected from images from 2017 to avoid overlapping with the development dataset. We collected PXRs with 25 femoral neck fracture, 25 trochanteric fracture, and 50 without hip fracture.

- 1. **Annotation of the development and testing dataset**

The ground truth label for the development and the testing dataset was reviewed by a 10-year experience trauma surgeon with clinical diagnosis, and surgery reports if the patient received surgery, advanced image modality such as CT scan, and radiologist report. Therefore, to determine the label does not rely on the PXR image alone.

1. **Algorithm development**
   1. **Platform and hardware**

The deep convolution neural network (DCNN) algorithm was developed on the TensorFlow 1.5.1[1] with Keras 2.1.4[2] open-source library under Python 3.6.5[3](Python Software Foundation). The whole development process was completed on an Intel® Core™ i7-7740X CPU 4.30 GHz with GeForce® GTX 1080 Ti GPU.

- 1. **Image preprocessing and augmentation**

All the images were downsized to 512 x 512 pixels. During the training process, the image augmentation methods were applied to amplify the image number, including horizontal flip, vertical flip, random rotation within 10 degrees, and random zooming with 10%.

- 1. **Neural network architecture selection**

We randomly separate the development dataset into 60% training, 20% validation, and 20% testing for initial evaluation of each neural network performance on the hip fracture classification. We tried VGG16[4], ResNet152[5], InceptionV3[6], InceptionResNetV2[7], and DenseNet-121[8] with binary classification with randomly initialized weight. The InceptionV3 models were easily overfitted, and the performance of testing set on the other network structures was poor. The DenseNet-121 model showed balanced performance regarding training, validation, and testing set. Therefore, DenseNet-121 was selected as the DCNN structure for further development.

- 1. **The training process of the algorithm**

The limb dataset was used as a pre-trained dataset. The DCNN was trained to class the limb X-ray into five body parts includes ankle, foot, knee, elbow, and wrist. The limb dataset was separated into 90% training and 10% validation in the training process with 60 epochs. The training accuracy achieved 99.5% on the body part classification task.

The fully connected layer of the DCNN was replaced for the hip fracture binary classification task. The PXR development dataset was separated into 80% training, 20% validation for the training process. We applied Adam[9] as an optimizer with the initial learning rate of 10^-3^. The batch size was eight and the DCNN was trained with 60 epochs without early stop. We used grad-CAM as a visualization algorithm for fracture site localization and also evaluate the model performance on hip fracture detection.

**Supplementary references**

1. Abadi M, Agarwal A, Barham P, Brevdo E, Chen Z, Citro C, et al. Tensorflow: Large-scale machine learning on heterogeneous distributed systems. arXiv preprint arXiv:160304467. 2016.

2. Chollet, Fran, et al. Keras. <https://kerasio2015>.

3. Team PC. Python: A dynamic, open source programming language. Python Software Foundation. 2015;78.

4. Simonyan K, Zisserman A. Very deep convolutional networks for large-scale image recognition. arXiv preprint arXiv:14091556. 2014.

5. He K, Zhang X, Ren S, Sun J, editors. Deep residual learning for image recognition. Proceedings of the IEEE conference on computer vision and pattern recognition; 2016.

6. Szegedy C, Vanhoucke V, Ioffe S, Shlens J, Wojna Z, editors. Rethinking the inception architecture for computer vision. Proceedings of the IEEE conference on computer vision and pattern recognition; 2016.

7. Szegedy C, Ioffe S, Vanhoucke V, Alemi AA, editors. Inception-v4, inception-resnet and the impact of residual connections on learning. Thirty-first AAAI conference on artificial intelligence; 2017.

8. Huang G, Liu Z, Van Der Maaten L, Weinberger KQ, editors. Densely connected convolutional networks. Proceedings of the IEEE conference on computer vision and pattern recognition; 2017.

9. Kingma DP, Ba J. Adam: A method for stochastic optimization. arXiv preprint arXiv:14126980. 2014.
